# HEARING LOSS AND INCIDENT DEMENTIA OVER 7 YEARS IN BLACK AND WHITE OLDER ADULTS IN ARIC-NCS

**DOI:** 10.1093/geroni/igac059.945

**Published:** 2022-12-20

**Authors:** John Shin, Kening Jiang, Nicholas Reed, David Knopman, Thomas Mosley, Richey Sharrett, Frank Lin, Jennifer Deal

**Affiliations:** Johns Hopkins Bloomberg School of Public Health, Baltimore, Maryland, United States; Johns Hopkins Cochlear Center for Hearing and Public Health, Baltimore, Maryland, United States; Johns Hopkins Bloomberg School of Public Health, Baltimore, Maryland, United States; Department of Neurology, Mayo Clinic, Rochester, Minnesota, United States; UMMC-The MIND Center, Jackson, Mississippi, United States; Department of Epidemiology, Johns Hopkins Bloomberg School of Public Health, Baltimore, Maryland, United States; Johns Hopkins Cochlear Center for Hearing and Public Health, Baltimore, Maryland, United States; Johns Hopkins University, Baltimore, Maryland, United States

## Abstract

Hearing loss is a risk factor for dementia; whether this association differs by race is unknown. Although hearing loss is less prevalent in Blacks than Whites, we hypothesized the hearing loss-dementia relationship is stronger in Blacks. All-cause dementia and mild cognitive impairment (MCI) were adjudicated using longitudinal cognitive data. Hearing was measured using pure tone better-ear thresholds (0.5-4 kHz). Cox proportional hazards models adjusted for demographic and clinical covariates and included an interaction term between hearing and race. In 3,605 participants from a population-based cohort (68-89 years, 23% Black, 59% female), estimates for 7-year dementia risk per 10 decibels increase in hearing loss were stronger in Blacks (hazard ratio [HR]:1.24, 95% Confidence Interval (CI):1.07,1.43) than Whites (HR:1.07; 95%CI:0.96,1.19). Hearing loss is a risk factor for dementia in Black Americans. These findings emphasize the need to address existing racial disparities in hearing healthcare.

